# A State Board of Health for Texas

**Published:** 1889-03

**Authors:** 


### EDITORIAL DEPARTMENT.

F. E. DANIEL, M. D., EDITOR AND PUBLISHER.

THIS JOURNAL, by unanimous vote of the members, was made the Official Organ of the Austin District Medical Society.

COLLABORATORS:

*Dr. R. M. Swearingen, Austin.*
*Prof. B. E. Hadra, M. D., Galveston.*
*Prof. Geo. Cupples, M. D., San Antonio.*
*Dr. T. C. Osborn, Cleburne, Texas.*
*Dr E. J. Doering, Chicago.*
*Dr. E. J. Beall, Fort Worth.*
*Dr Odo Betz, Germany.*
*Dr. J. W. McLaughlin, Austin.*
*Dr. T. J. Tyner, Austin.*
*Prof. J. F. Y. Paine, M. D., Galveston.*
*Dr. R. H. L. Bibb, Mexico.*
*Dr. T. J. Bennett. Austin.*
*Dr. Bat Smith, Wharton.*
*Dr. E. Meierhof, New York.*
*Prof. W. B. Rogers, M. D., Memphis, Tenn.*

### A STATE BOARD OF HEALTH FOR TEXAS.

*Parturiunt Montes, Etc.*

The profession in Texas have never agreed precisely as to what is needed in the way of a Board of Health. In a vague sort of a way there is a general desire that Texas should have a State Board because other states have them ; that, we believe, is the principal reason assigned for the immediate creation of a Board. And we may say, the majority of the physicians would favor the passage of an act creating a good and efficient Board, should they be asked to do so. Bnt, as stated, although there have been frequent consultations held on the subject, and it has been discussed in the meetings of the Texas State Medical Association, there has been no definite outcome in the way of a bill for the creation of a Board, emanating from this source. That is to say, not since the rejection by the immortal 19th Legislature, of the bill adopted by the Association at Belton in 1884.

That the request for a Board of Health should emanate from the State Medical Association in order to reflect the views of the profession at large in Texas would seem eminently right and proper. The Association is distinctly representative of the whole profession of the State, being composed of one or more delegates from every county in the State, in addition to delegates from all the local and district medical organizations. These delegates represent the views of their constituents on all matters pertaining to medical and sanitary science, and in order to get at the wishes or views of the physicians throughout the State, it is necessary that the delegates shall be canvassed. In view of the great diversity of opinion held on this, as on all subjects by Doctors, the State Association thought best to assign it to a selected committee, called the Committee on Medical Legislation, leaving the matter entirely to their judgment and discretion, as to how and when to ask, and for what legislation, or to ask not at all. This committee was chosen with especial reference to the wisdom, experience and sound opinions of its members on this particular subject, it being one to which they had given much attention and careful reflection. The chairman is the venerable Dr. George Cupples—the "Nestor of Texas Surgery," as full of honors as of years. His colleagues are Drs. Wooten, McLaughlin, Tyner, of Austin, Clopton, of Jefferson, Pope, of Marshall, and Kelley, of Galveston. This committee, it will be seen, represents Texas, in the north, east, south, west and centrally. To date, these gentlemen, so far as we know, have never held a meeting on the subject; certain it is that they have formulated no bill for the creation of a State of Board of Health, and have made no move whatever as a committee, in the direction of asking for one. What are we to infer from this? Undoubtedly, that in their opinion public sentiment is not yet ripe for a change from the present so called health system (quarantine) which, having effectually kept out pestilence, has satisfied the public;—in other words—the time for action has not arrived; or else, that they have not succeeded in collating the views of their constituents on the subject, without which, no intelligent draft of a law could be formulated which would unquestionably reflect the sentiment of the profession at large. The matter has been left entirely to them, and they have not acted. What other construction is to be placed upon it?

Meantime, however, certain patriotic individuals, more ambitious to serve their country and protect the dear people (who have not asked for it) than to fnrther their own ends, no doubt, have plied the legislature with bills and amendments, asking for a Board of Health on their own hook, as it were; not content with the ox-cart ways of the sober and wise committee, to whose judgment some five hundred delegates, representing the five thousand physicians at large saw proper to leave it; impatient of delay, they have moved heaven and earth in a small way (a kind of a tempest in a teapot), to rush through a bill which will abolish the present system, and create a cumbersome machine of seven to fifteen parts, each of which would have to be consulted, in as many different parts of the State, before an embargo could be laid on a car load of infected goods halted at Sabine or El Paso. In other words, would assume, unasked, the duty assigned to others.

Under the circumstances the "assistance" thus tendered the committee is a little previous, if not a species of down right impertinence. They have asked for no suggestions, and certainly require no volunteer aid in the draft of a bill, even should they have decided upon one; and we may say, they need no suggestions as to their especial office. When they do, doubtless they will ask for it. The bill having been prepared ready to the hands of the committee, they have been importuned to adopt it; but so far, they have, as a committee, left it severely alone, and have given members to understand that it is not their affair. The medical profession of Texas know nothing of the movement, except by newspaper report; they have not been consulted, and have had no hand in the matter; and therefore, it does not reflect the medical sentiment of the State, notwithstanding the local support given it by the signatures of a score or so of reputable physicians.

Well, it will not pass; the Senate Committee have reported favorably upon it, but it has gotten no further, and will halt where it is until adjournment, notwithstanding the immense (?) personal lobbying brought to bear in its interest. The Legislature already "smell a mice;" they see how the land lies, and doubtless have concluded that when the medical profession of Texas, and not of one county alone, want a board of health, they will ask for it, and present a bill which will need no doctoring. The regular session of the Legislature is over, and the members being now on less than half pay ($2 a day) are restless and anxious to get away. Moreover, each one has some pet scheme to further, and he is bending all his energies in the direction of bringing it up, before the close, and it will be extremely difficult to enlist their attention in any new business.

A State Board of Health is a question of the future, and the present bill is a dead letter.

